# Does Intrauterine Exposure to Diabetes Impact Mental and Motor Skills? A Meta-Analysis of the Bayley Scales of Infant Development

**DOI:** 10.3390/ijerph21020191

**Published:** 2024-02-07

**Authors:** Diana Arabiat, Mohammad AL Jabery, Lisa Whitehead

**Affiliations:** 1 Maternal and Child Nursing Department, School of Nursing, The University of Jordan, Amman 11942, Jordan; l.whitehead@ecu.edu.au; 2 The Centre for Evidence Informed Nursing, Midwifery and Healthcare Practice, Joondalup 6027, Australia; 3 School of Nursing and Midwifery, Edith Cowan University, Joondalup 6027, Australia; 4 Department of Special Education, School of Educational Sciences, The University of Jordan, Amman 11942, Jordan; m.algabery@ju.edu.jo

**Keywords:** mental development, psychomotor development, maternal diabetes, pregnancy, Bayley scales, BSID, child development

## Abstract

Background: Attempts to conduct meta-analyses of the association between child development and diabetes have been limited by the wide range of tools and definitions of developmental outcomes used in the literature. We aim to meta-analyze a widely used measure of child development, the Bayley Scales of Infant Development, with respect to exposure to diabetes and developmental scores. Methods: PsycINFO, MEDLINE/PubMed, EMBASE, Emcare, and Google Scholar databases were searched. Two independent reviewers screened, extracted, and quality-appraised the studies using JBI SUMARI software. Forest plots were created with the standardized mean difference using the random-effects model, and heterogeneity was assessed using *I*^2^. Results: Seven studies were identified. The pooled results on psychomotor and mental development index mean scores were lower for infants born to mothers with diabetes than for the control group (Cohen’s d = −4.49, df = 7, *I*^2^ = 0%, *p* = 0.001 and Cohen’s d = −3.4, df = 9, *I*^2^ = 27%, *p* = 0.001, respectively). Effects were larger in infants born to mothers with type 1 and 2 diabetes and at age 12 months. Conclusions: Maternal diabetes should be considered as a risk factor for children’s development, mainly when born to mothers with pre-existing diabetes.

## 1. Introduction

Psychomotor performance is the set of skills that children acquire in the first five years of life, comprising cognitive, emotional, motor, and social capacities [1]. In some situations, children may experience a delay or slow progress in the attainment of one or more developmental domains, and this is called psychomotor retardation or development delay [2]. A delay in psychomotor development can be related to numerous risk factors, including premature birth, low birth weight, or intrauterine growth restriction [3]. Earlier research has found a strong association between child development and intrauterine exposure to diabetes, though the findings are limited by the use of cross-sectional design, variability in definition of child development, and the types of diabetes explored, as there has been a focus on some types more than others. Risk factors, including obesity and low socio-economic status [4,5], create additional confounders when studying the impact of intrauterine exposure to diabetes.

A recent review by Rodolaki et al. [5] suggested that intrauterine exposure to hyperglycemic injury when combined with obesity can predispose children to mood abnormalities and intellectual impairment. The authors stated that despite conflicting findings on diabetes’s impact on the child’s brain, the neurocircuitry of the child is often disrupted by intrauterine hyperglycemia that may exaggerate peripheral inflammatory response and neuroinflammation in the brain.

Moreover, there have been several informative systematic reviews surrounding the association between intrauterine exposure to diabetes and cognitive [6,7], language [7], and motor development in children [4]. However, the reviews are limited by considerable variability in the operationalization of child development and a high degree of heterogeneity, limiting comparisons between studies. Variation in the definition of child development and developmental delay will influence the report of outcomes, and there is value in prioritizing a meta-analysis of studies that use a common measure to assess child development. Therefore, we sought to analyze the association between intrauterine exposure to diabetes and child development by analyzing studies that employed the Bayley Scales of Infant Development (BSID) [8,9], the Psychomotor Developmental Index (PDI), and the Mental Developmental Index (MDI) with infants up to the age of 24 months.

The Bayley Scales of Infant Development is a widely used measure of functionality of typically developing and high-risk infants. BSDI-I and BSID-II comprise two scales including the Mental Developmental Index (MDI) and Psychomotor Development Index (PDI). The MDI provides an assessment of memory, problem solving, sensory perception, hand–eye coordination, imitation, and early language, while the PDI measures gross and fine motor skills. BSDI-III comprises additional scales of a cognitive, language, and behavioral composite. Each of the three editions of the BSID differ in scaling and age range. For example, BSID-I can be used in assessing infants aged between 2 and 30 months, while BSID-II and -III can be used among infants aged 1 month to 42 months. Normed scores of the three editions have a mean of 100 and a standard deviation of 15, with higher scores reflecting better development.

## 2. Materials and Methods

This review has been conducted in accordance with the Johnna Briggs Institute (JBI) methodology for systematic reviews [10] and following the Preferred Reporting Items for Systematic Review and Meta-Analysis statement extension for network meta-analysis. The protocol was registered with the International Prospective Register of Systematic Reviews (PROSPERO registration # CRD42021282758).

### 2.1. Eligibility Criteria, Literature Search, and Study Selection

A systematic search was conducted from the database inception to August 2023 to identify all studies reporting the developmental outcomes of children born to mothers with and without diabetes. Only studies that reported means and standard deviations were included. Of all developmental outcomes, only those studies reporting by the Bayley Mental Developmental Index (MDI) and Bayley Psychomotor Developmental Index (PDI) were eligible for inclusion.

The following electronic databases were searched using a comprehensive search strategy developed by a librarian: the PsycINFO, MEDLINE/PubMed, EMBASE, Emcare, and Google Scholar databases. First, we conducted computer-based searches using PubMed and Ovid for the following terms (or stems when appropriate) appearing anywhere in the manuscript: (diabetes) AND (BSID OR “Bayley Scales of Infant Development”) OR (PDI OR “Psychomotor Development Index”) OR (MDI OR “Mental Development Index”). Second, we reviewed the titles and abstracts of relevant articles and the index terms used to describe the articles for additional studies using forward and backward searching.

Second, following the initial search, all identified citations were collated and uploaded into Rayyan, an online systematic review software [11], and duplicates were removed. Third, titles and abstracts were screened by two reviewers independently against the inclusion criteria for the review. Discrepancies and disagreements were jointly resolved by the reviewers and in consultation with the first study author.

### 2.2. Outcomes

The main outcomes were the mental development and psychomotor development scores of infants born to mothers with diabetes and to mothers without diabetes.

### 2.3. Data Extraction and JBI Quality Appraisal

Data for each eligible study were extracted by two independent reviewers and compared to ensure accuracy. All data related to mental and psychomotor development scores were extracted and input into the JBI System for the Unified Management, Assessment, and Review of Information (SUMARI) software. Extracted data included information about the exposure of interest, populations, methods, and outcomes. Quality assessment of eligible studies was evaluated using the relevant JBI Critical Appraisal Checklist for Systematic Reviews and Research Synthesis. Studies were considered of poor quality if a score of 50% or less was assigned through methodological quality.

### 2.4. Statistical Analysis

A series of pairwise conventional meta-analyses were performed using JBI SUMARI [12] to assess for associations between intrauterine exposure to diabetes and study outcomes. We used random-effects models using inverse variance weighting to obtain pooled estimates of SMD and the corresponding 95% CI. A random-effects model was used since it is assumed that studies were estimating the effects of diabetes in different populations across multiple types of maternal diabetes. Our first set of the meta-analysis included the data from all studies to obtain an overall pooled estimate and to examine whether there was heterogeneity in results between studies. Means, standard deviations (SDs), and sample size for the group of infants of mothers living with diabetes and controls were used to calculate effect size (Cohen’s d) or standardized mean differences (SDM) for both the MDI and PDI scales. An estimate of 0.2 for the SMD effect size was considered small, 0.5 as moderate, and 0.8 as large. The 95% confidence interval (CI) for the effect size represents the relative precision of the measurement. A wider range suggests the results are less precise.

If a study reported multiple types of diabetes and times of measurement, we calculated the combined mean and SD. For studies reporting the type of diabetes, we performed specific subgroup analyses to examine whether the difference in development outcomes between the diabetes group and control group were influenced by the type of maternal diabetes or the child’s age at time of assessment. Finally, heterogeneity between studies was quantified using the Cochran Q and *I*^2^ statistics. Statistical significance was defined as a *p*-value *<* 0.10 for the Q test and a value *>* 50% for the *I*^2^.

## 3. Results

**Figure 1:**
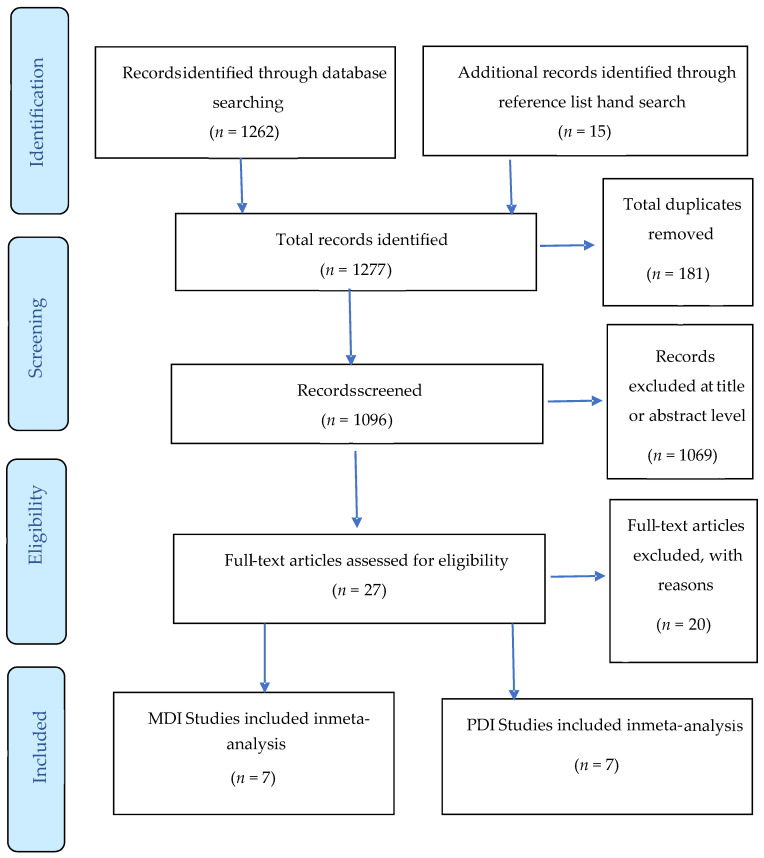
Preferred Reporting Items for Systematic Review and Meta-analysis (PRISMA) Flowchart of literature and selection Process.

As shown in the PRISMA Figure 1, the database searches yielded 1277 studies, of which 181 were excluded on the basis of duplication and 1069 were excluded after screening the title or abstract. We assessed the full text of the remaining 27 studies. We excluded 20 studies as they did not meet the inclusion criteria by age or did not use the BSID to report developmental outcomes. The remaining 7 records were reviewed in detail and categorized according to type of diabetes and type of developmental domain (MDI vs. PDI).

### 3.1. Study Characteristics

Every study included in the meta-analysis and the assessed variables are presented in Table 1. Two studies used medical records to confirm the diagnosis of diabetes [13,14], and one study [15] used criteria for gestational diabetes of fasting glucose 5.1 mmol/L, 1 h result 10.0 mmol/L, or 2 h 8.5 mmol/L. The remaining studies lacked information about the criteria used to confirm diabetes.

In three studies, infants born to mothers with diabetes were compared to controls without specifying the underlying type of diabetes [16,17,18]. In Sells et al. [19], infants born to mothers with insulin dependent diabetes were compared to controls. Comparison between pre-existing and/or gestational diabetes was made in four studies [14,15,16,20]. Two studies [14,15] matched participants with diabetes and controls for age, gender, and other demographic factors. Few studies adjusted their analyses by other potential confounding factors such as gestational age [16,17] and/or maternal age [16,20]. Reports of confounding factors such as body mass index (BMI) varied across studies, and only one study [20] presented an analysis according to the weight of the mother (see Table 1).

**Table 1:** Characteristics of studies on developmentally relevant behavior and mental health of children born to mothers with and without diabetes.

Authors, Year (Design, JBI Quality Score)	Country	Type of Maternal Diabetes and Criteria for Diagnosis	Age in Months	Domain of Development	Mean and SD on Developmentally Relevant Mental and Psychomotor Measure			*p* Value	Variable Controlled for	Cohen’s d
					*N*	*M*	*SD*			
deRegnier et al. [17] (Case control, 9/10)	USA	Diabetes	12	MDI of BSID-II	22 IDM	102.6	5.6	NS	Adjusted for prematurity	0.39
					27 Control	104.7	5.2			
				PDI of BSID-II	22 IDM	100.6	3.4	NS		0.87
					27 Control	103.4	1.9			
He et al. [16] (Cohort, 10/11)	China	GDM (GDM was diagnosed if fasting glucose 5.1 mmol/L, or if the 1 h result 10.0 mmol/L, or the 2 h result 8.5 mmol/L).	12	MDI of BSID-I	177 GDM	123.21	13.78	0.023	Adjusted for maternal age, gestational age, saturated fatty acid, FBS, and neonatal weight	0.21
					378 Control	126.06	13.73			
				PDI of BSID-I	177 GDM	110.59	15.16	0.017 *		0.22
					378 Control	113.81	14.56			
Hod et al. [15] (Cohort study, 9/10)	Israel	PDM (Known cases from medical records)	12	MDI of BSID-II	31 PGDM	91.04	9.01	<0.05	Matched for age, education, religious belief, and ethnic origin	0.66
					41 Control	98.15	12.05			
				PDI of BSID-II	31 PGDM	85.15	14.53	<0.05		0.63
					41 Control	95.54	18.14			
Levy-Shiff, et al. [14] (Case control, 9/10)	Israel	PDM, GDM (Medical records)	12	MDI of BSID-II	53 PGDM	91.04	9.01	<0.05	Not adjusted, but matched for demographic criteria	0.71
					51 GDM	92.09	12.09			0.53
					49 Control	98.53 *	12.05			
					Combined diabetes group	91.6	10.6			0.67
				PDI of BSID-II	53 PGDM	85.15	14.53	<0.05		0.63
					51 GDM	94.15	13.86			0.09
					49 Control	95.54 *	18.14			
					Combined diabetes group	89.6	14.8			0.36
Nelson et al. [18] (Case control, 8/10)	USA	Diabetes (Fetal hyperinsulinemia was assessed chronically by neonatal macrosomia, neonatal hypoglycemia, elevated neonatal hemoglobin, and low neonatal serum ferritin)	12	MDI of BSID-II	25 IDM	103	7.5	NS	Not adjusted	0.24
					32 Control	105	8.7			
				PDI of BSID-II	25 IDM	102	9.5	NS		0.0
					32 Control	102	11.9			
Sells et al. [19] (Cohort study, 9/11)	USA	Insulin-dependent diabetes	6	MDI of BSID-I	51 Early entry	104	14.4	NS		
					32 Late entry	106	13.7			
					84 Control infants	107	12.1			
					Combined diabetes group	105.8	14.1			0.17
			12		62 Early entry	113	15.3	NS		
					31 Late entry	112	13.5			
					83 Control infants	117	12.5			
					Combined diabetes group	112.7	14.7			0.31
			24		49 Early entry	118	19.4	NS		
					22 Late entry	112	16.8			
					78 Control infants	118	18.4			
					Combined diabetes group	116.1	18.7			0.11
			6	PDI BSID-I	51 Early entry	109	16.2	NS		
					32 Late entry	104	17.0			
					83 Control	108	15.0			
					Combined diabetes group	107.1	16.6			0.06
			12		62 Early entry	104	15.5	NS		
					31 Late entry	102	17.2			
					83 Control	103	15.8			
					Combined diabetes group	103.3	16			0.02
					47 Early entry	108	17.5	NS		
			24		22 Late entry	112	21.5			
					78 Control	110	18.2			
					Combined diabetes group	109.3	18.8			0.04
Torres-Espinola et al. [20] (Case control, 10/10)	Spain	GDM (groups based on their calculated pre-gestational BMI and their gestational diabetes status)	6	MDI of BSID-III	81 Normal weight BMI < 25 kg	101.1	15.6	NS	Maternal age, maternal education, placental weight, and weight gain during pregnancy	0.11
					44 Overweight BMI < 30 kg	102.2	16.5			
					32 Obese BMI ≥ 30 kg	94.4	18.7			
					58 GDM	99.4	13.6			
			18		75 Normal weight	102.8	15.2	NS		0.05
					43 Overweight	102.8	17.8			
					29 Obese	98.1	17.1			
					50 GDM	102.1	15.8			
			6	PDI BSID-III	81 Normal weight	105.8	11.2	NS		0.18
					44 Overweight	104.5	11.1			
					32 Obese	106.6	11.1			
					58 GDM	103.7	12.6			
			18		75 Normal weight	118.1	10.2	NS		0.34
					43 Overweight	115.4	8.0			
					29 Obese	114.1	10.0			
					50 GDM	115.0	7.5			

GDM: gestational diabetes; PDM: pre-existing diabetes; MDI: Mental Development Index; PDI: Psychomotor Development Index; BSID-II: Bayley Scale of Infant Development (version II), NS: not significant (*p* > 0.05). * *p* ≤ 0.05.

### 3.2. Developmental Outcomes

Seven studies generated 10 data entries that could be included in the meta-analysis of mental and psychomotor development outcomes as measured by the Bayley scales. For each study, we calculated the effect size by the type of diabetes and the age of the infant at time of assessment.

Four studies [17,18,19,20] reported no significant difference on mental or psychomotor development scores between the groups. However, in three studies, children born to mothers with diabetes were reported to have significantly lower scores on the MDI [14,15,16]. Levels of fructosamine were associated with lower scores in the children born to mothers living with diabetes on the MDI and PDI, as well as the levels of HbA1C and PDI scores [14]. In Torres-Espinola et al. [20], exposure to gestational diabetes in obese and overweight mothers was suggested as a risk of motor development; however, the authors suggest caution in the interpretation of the findings where their results were not significant when confounders were adjusted for.

Developmental outcomes in most studies were evaluated primarily at age 12 months using BSID-II. BSID-I was used in four studies to assess motor and mental domains in the age groups from 3 to 28 months, while the BSID-II published in 1993 and the BSD-III published in 2006 included the addition of a behavior rating scale and assessment of the age group 1 to 42 months. Mental and psychomotor developments were measured infrequently before or after 12 months, and limited data were reported on the behavior domain. The meta-analysis demonstrated that psychomotor development in children born to mothers living with diabetes was significantly impaired.

### 3.3. Meta-Analysis of Bayley MDI

**Figure 2:**
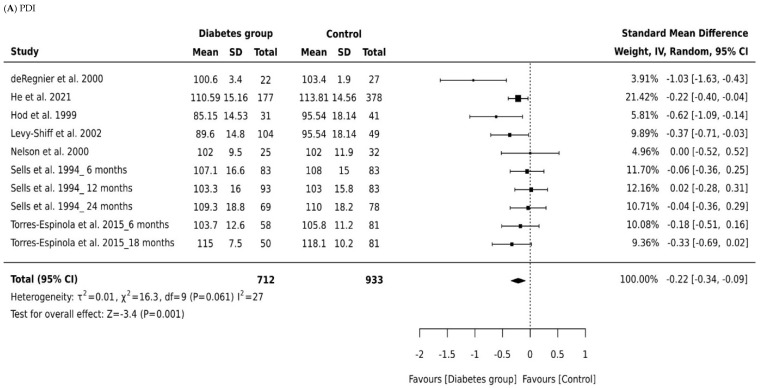

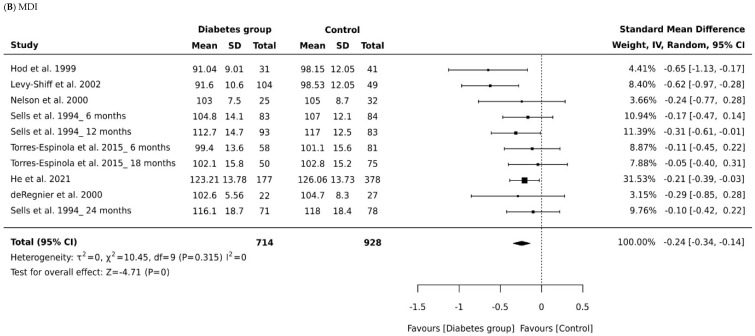
Forest plots comparing differences in: (**A**) Psychomotor Development Index (PDI) scores of infants born to mothers with and without diabetes (References mentioned respectively [14,15,16,17,18,19,20]) and (**B**) Mental Development Index (MDI) scores of infants born to mothers with and without diabetes (References mentioned respectively [14,15,16,17,18,19,20]).

A total of 1642 infants were included across all studies that measured MDI, with 714 children born to mothers with diabetes and 928 controls. Figure 2A depicts a forest plot of the MDI scores by group across the seven studies, grouped according to type of diabetes and the age of the child at time of assessment. The pooled analyses (Table 2) combining all cohorts demonstrated a small effect for intrauterine diabetes exposure on MDI (Cohen’s d = −0.24, 95% CI −0.34, −0.14, *p* = 0.001) when compared to controls. There was minimum heterogeneity in results between studies: the p value for the Q-test for heterogeneity beyond chance was insignificant, and *I*^2^ was 0%. The significant difference in pooled effect size between the children born to mothers living with diabetes and controls remained significant in all subgroup analysis of data for children born to mothers with gestational diabetes and infants born to mothers with pre-existing diabetes (pre-existing diabetes, Cohen’s d = −0.21, 95% CI −0.34 to −0.07; gestational diabetes, Cohen’s d = −0.51, 95% CI −0.91 to −0.07).

We analyzed also whether effect size in MDI scores between the children born to mothers with diabetes and controls depended on the child’s age at the time of assessment. Only two studies were identified as reporting appropriate data for the MDI meta-analysis and focused primarily on children aged 6, 12, and 18 months in Torres-Espinola et al. [20] and on children aged 6, 12, and 24 months in Sells et al. [19]. Those studies produced a nonsignificant effect size for MDI in children aged 6 months and 18 months or older (Cohen’s d = −0.10, 95% CI, −0.33, 0.12, *p* = 0.0.379; Cohen’s d = −0.08, 95% CI, −0.32, 0.16, *p* = 0.0.53; Figure 2, Table 2). Thus, maternal diabetes was only associated with decreased MDI scores in children aged 12 months (Cohen’s d = −0.034, 95% CI, −0.46, −0.22, *p* = 0.001). There was some evidence of heterogeneity associated with effect size for the MDI scores at age 12 months (*I*^2^ = 32%). An overall effect size of −0.08 was observed for all types of diabetes across all outcome measures of the MDI. Our findings show mean effect sizes were higher for children born to mothers with pre-existing diabetes than for children born to mothers with gestational diabetes.

### 3.4. Meta-Analysis of Bayley PDI

A total of 1645 children were included in all studies that measured PDI, with 712 children born to mothers with diabetes and 933 controls. The effect size of the PDI relative to controls is shown in Figure 2B. There was a small significant difference in pooled effect size between the outcomes for children born to mothers with diabetes and controls (Cohen’s d = −0.22, 95% CI −0.34 to −0.09, P = 0.001). There was minimum heterogeneity in results between studies: the *p* value for the Q- test for heterogeneity beyond chance was 0.0001, and *I*^2^ was 27%. Significant differences were noted for both children born to mothers with gestational diabetes (Cohen’s d = −0.20, 95% CI −0.36 to −0.03) and pre-existing diabetes (Cohen’s d = −0.62, 95% CI −0.93 to −0.32). The effect size of pre-existing diabetes was stronger, suggesting a higher negative impact of type 1 and type 2 diabetes compared to gestational diabetes. However, we suggest caution in interpreting this finding due to the small numbers of studies included in the subgroup analyses.

The age of the child at time of assessment was not directly associated with the MDI or PDI score among infants aged 18 months or older (Cohen’s d = −0.17, 95% CI, −0.41 to 0.07, *p* = 0.167: Figure 2, Table 2). Age did, however, influence psychomotor development scores for children aged 6 months (Cohen’s d = 0.44, 95% CI, 0.20 to 0.67, *p* = 0.0001). Infants born to mothers living with diabetes at age 12 months had significantly lower PDI scores than those born to mothers without diabetes (Cohen’s d = −0.36, 95% CI, −0.58 to −0.14, *p* = 0.0001). Our data indicate that children born to mothers with diabetes have PDI scores within the typical range; in other words, they “catch up” in the areas of mental and psychomotor development. A large effect size was found for the association between pre-existing diabetes and PDI scores. Effect sizes were also higher in studies of infants aged 12 months in comparison to studies of infants aged 6 months and 18 months and older.

## 4. Discussion

Seven studies were included in the meta-analysis. Combining all available data, we found significant differences in both MDI and PDI scores between children born to mothers with diabetes and controls. Results were homogeneous, indicating minimum differences existed between subgroups for the overall analysis. This finding is consistent with recent reviews [5] and a pairwise conventional meta-analysis [4,8] that suggested intrauterine exposure to diabetes was associated with decreased scores for both fine and gross motor abilities [4] and lower expressive language development [8] compared to controls. The results of those reviews also raise the question of whether types of diabetes and other comorbidities, such as overweight and obesity, should be considered as additional moderating variables with potentially different clinical and physiological effects.

Subsequent explanatory subgroup analyses by type of diabetes revealed that studies assessing child development reported significantly lower mental and psychomotor development scores in children born to mothers with pre-existing diabetes compared with controls with no previous exposure to diabetes. Lower mental and psychomotor development scores were also reported for children born to mothers with gestational diabetes compared with controls. One important finding that emerged from our synthesis was that the effect size for measures of both mental and psychomotor development scores were higher when studies included children born to mothers with pre-existing diabetes. The effect size was higher for both mental and psychomotor development scores when studies included MDI scores for infants aged 12 months only. This suggests that using terms, such as ‘developmentally delayed’ or ‘impaired development’, for describing the development of infants of diabetic mothers may be an inappropriate characterization since the results of the subgroup meta-analysis suggest these infants will exhibit “developmental spurts” and catch up with their peers at age 18 months and older. In addition, only one study [15] reported a standardized mean score lower than 85 for children born to mothers with diabetes, the cut-off score for diagnosing developmental delay. Overall, the standardized mean score was 100 (SD = 15), with scores lower than 85 indicating mild developmental delay, and a score lower than 70 indicating moderate or severe developmental delay.

Earlier reviews [5,8,21,22] have shown that intrauterine exposure to diabetes impacts child development through an increase in insulin resistance, giving rise to hyperglycemia that may be associated with hyperketonemia, potentially leading to hypoxia and increased metabolism of the brain. Children born to mothers with pre-existing diabetes may also have an altered fuel environment that may lead to fuel-mediated teratogenicity via a direct toxic effect of high glucose concentrations impacting the development of brain cells, pancreatic beta cells, adipose and muscle cells, and nephrons and with long neurodevelopmental consequences for the unborn child [5,23].

Finally, the results from this meta-analysis are consistent with the literature that suggests intrauterine exposure to pre-existing diabetes negatively influences child development. Maternal comorbidity and glycemic control variables, such as HbA1C and hypoglycemia, in children played an important role in their early development. Therefore, future research needs to consider the impact of maternal diabetes on child development within a larger context of interdependent elements and processes such as maternal comorbidity, parent and child relations, and socio-economic status. What factors, for instance, mediate the relationship between maternal diabetes and child development? What kinds of environmental and social factors relate to maternal diabetes and impact on children’s overall development? Studies that differentiate between type of diabetes and level of glycemic control need to be conducted. Children born to mothers with pre-existing diabetes may experience greater developmental challenges than children born to mothers with gestational diabetes. Analysis that combines diabetes by type may fail to identify differences that exist within samples.

## 5. Limitations

By using a single assessment measure for the developmental outcomes (the BSID), our meta-analysis maximized consistency of the measurement of child development. It further increases confidence in the effect-size estimates in relation to diabetes. Limiting our studies to those that used the BSID allowed the exploration of associations between diabetes and child development, with a focus on specific types of development (psychomotor vs. mental development) measured at the same time point, using the same scale.

An additional strength of this review was the inclusion of studies that were mostly homogeneous in nature, mainly in relation to type of diabetes, age at follow-up, assessment tools, and follow-up time; however, some heterogeneity was noted in the meta-analysis (Figure 2). It precluded the determination of whether sample size bias accounted for findings. Over half of the studies included in this meta-analysis used a cross-sectional design and had a small sample size, and as such, our conclusions should be met with caution. Our study was limited to follow-ups at age 18 and 24 months, and some differences in child development may not fully appear until late childhood [24]. However, maternal diabetes has been associated with lower scores on mental and psychomotor developments in children at age 12 months. Finally, specific attention was not directed to the role of gestational comorbidities, such as overweight, obesity, maternal depression, and the social context of the child.

## 6. Conclusions

In this meta-analysis, diabetes was found to be a crucial factor in mental and psychomotor development outcomes, in particular for children aged 12 months and under. Despite advancing maternal care, the results show a statistically small effect size of diabetes on child development. Because most of the studies did not consider the role of other comorbidities and confounding variables, it is important to consider that these may also play a role. There is a need for future studies with larger sample sizes to generate a reasonable degree of statistical power and allow for an increase in generalizability of the association between intrauterine exposure to diabetes and child development. Future studies must also consider including homogeneous groups of maternal diabetes, clear descriptions of moderating variables, such as BMI, values of glycemic control, and other socio-economic statuses to evaluate whether the effects of diabetes were maintained regardless of other confounding or moderating variables.

## Figures and Tables

**Table 2:** Meta-analysis results classified by type of diabetes and child age at the time of assessment.

			Number of Studies	Sample Size		Cohen’s d (SMD)	*p*	95% CI	*I* ^2^	*p*
				Diabetes Group	Control Group					
MDI										
	Type of diabetes									
		GDM	3	336	583	−0.21	0.003 ***	−0.34, −0.07	0%	0.305
		PDM	2	84	100	−0.51	0.013 *	−0.91, −0.11	45%	0.179
	Child’s age									
		6 months	2	141	165	−0.10	0.379	−0.33, 0.12	0%	0.92
		12 months	6	452	655	−0.34	0.001 **	−0.46, −0.22	32%	0.195
		>12 months	2	121	153	−0.08	0.531	−0.32, 0.16	0%	0.817
	All studies		7	714	928	−0.24	0.001 **	−0.34, −0.14	0%	0.315
PDI										
	Type of diabetes									
		GDM	2	228	427	−0.20	0.019 *	−0.36, −0.03	0%	0.548
		PDM	2	84	90	−0.62	0.001 **	−0.93, −0.32	0%	0.936
	Child’s age									
		6 months	2	141	164	0.44	0.001 **	0.20, 0.64	96%	0.001 **
		12 months	6	375	610	−0.36	0.001 **	−0.58, −0.14	45%	0.085
		>12 months	2	119	153	−0.17	0.167	−0.41, 0.07	31%	0.229
	All studies		7	712	933	−0.22	0.001 **	−0.34, −0.09	27%	0.061

* *p* < 0.05, ** *p* < 0.001, *** *p* < 0.005; GDM: gestational diabetes; PDM: pre-existing diabetes.

## Data Availability

The data used to support the findings of this review are available on request from the authors.

## References

[1] Pazera G., Młodawska M., Kukulska K., Młodawski J. (2023). The Assessment of Psychomotor Development in Full-Term Children at 12 Months of Age with Munich Functional Development Diagnostics Depending on the Feeding Method: A Cross-Sectional Study. Pediatr. Rep..

[2] Hadar-Frumer M., Ten Napel H., Yuste-Sánchez M.J., Rodríguez-Costa I. (2023). The International Classification of Functioning, Disability and Health: Accuracy in Aquatic Activities Reports among Children with Developmental Delay. Children.

[3] Demirci G.M., Kittler P.M., Phan H.T., Gordon A.D., Flory M.J., Parab S.M., Tsai C.L. (2023). Predicting mental and psychomotor delay in very pre-term infants using machine learning. Pediatr. Res..

[4] Arabiat D., Jabery M.A., Kemp V., Jenkins M., Whitehead L.C., Adams G. (2021). Motor developmental outcomes in children exposed to maternal diabetes during pregnancy: A systematic review and meta-analysis. Int. J. Environ. Res. Public Health.

[5] Rodolaki K., Pergialiotis V., Iakovidou N., Boutsikou T., Iliodromiti Z., Kanaka-Gantenbein C. (2023). The impact of maternal diabetes on the future health and neurodevelopment of the offspring: A review of the evidence. Front. Endocrinol..

[6] Sukmakarti L.D., Murti B., Adriani R.B. (2023). Meta Analysis: Effects of Polycystic Ovarian Syndrome and Maternal Diabetes on the Risk of Autism in Children. J. Matern. Child Health.

[7] Camprubi Robles M., Campoy C., Garcia Fernandez L., Lopez-Pedrosa J.M., Rueda R., Martin M.J. (2015). Maternal diabetes and cognitive performance in the offspring: A systematic review and meta-analysis. PLoS ONE.

[8] Arabiat D., Jabery M.A., Jenkins M., Kemp V., Whitehead L., Adams G. (2021). Language abilities in children born to mothers diagnosed with diabetes: A systematic review and meta-analysis. Early Hum. Dev..

[9] Munn Z., Aromataris E., Tufanaru C., Stern C., Porritt K., Farrow J., Lockwood C., Stephenson M., Moola S., Lizarondo L. (2019). The development of software to support multiple systematic review types: The Joanna Briggs Institute System for the Unified Management, Assessment and Review of Information (JBI SUMARI). JBI Evid. Implement..

[10] Bayley N. (1969). Bayley Scales of Infant Development.

[11] Bayley N. (1993). Bayley Scales of Infant Development.

[12] Ouzzani M., Hammady H., Fedorowicz Z., Elmagarmid A. (2016). Rayyan—A web and mobile app for systematic reviews. Syst. Rev..

[13] Piper C. (2019). System for the unified management, assessment, and review of information (SUMARI). J. Med. Libr. Assoc. JMLA.

[14] Levy-Shiff R., Lerman M., Har-Even D., Hod M. (2002). Maternal adjustment and infant outcome in medically defined high-risk pregnancy. Dev. Psychol..

[15] Hod M., Levy-Shiff R., Lerman M., Schindel B., Ben-Rafael Ζ., Bar J. (1999). Developmental outcome of offspring of pregestational diabetic mothers. J. Pediatr. Endocrinol. Metab..

[16] He X.J., Dai R.X., Tian C.Q., Hu C.L. (2021). Neurodevelopmental outcome at 1 year in offspring of women with gestational diabetes mellitus. Gynecol. Endocrinol..

[17] de Regnier R.A., Nelson C.A., Thomas K.M., Wewerka S., Georgieff M.K. (2000). Neurophysiologic evaluation of auditory recognition memory in healthy newborn infants and infants of diabetic mothers. J. Pediatr..

[18] Nelson C.A., Wewerka S., Thomas K.M., deRegnier R.A., Tribbey-Walbridge S., Georgieff M. (2000). Neurocognitive sequelae of infants of diabetic mothers. Behav. Neurosci..

[19] Sells C.J., Robinson N.M., Brown Z., Knopp R.H. (1994). Long-term developmental follow-up of infants of diabetic mothers. J. Pediatr..

[20] Torres-Espinola F.J., Berglund S.K., García-Valdés L.M., Segura M.T., Jerez A., Campos D., Moreno-Torres R., Rueda R., Catena A., Pérez-García M. (2015). Maternal obesity, overweight and gestational diabetes affect the offspring neurodevelopment at 6 and 18 months of age—A follow up from the PREOBE cohort. PLoS ONE.

[21] Cordero M.J.A., Garcia L.B., Blanque R.R., Garacia J.L., Villar N.M., Lopez A.M.S. (2015). Maternal diabetes mellitus and its impact on child neurodevelopment; systematic review. Nutr. Hosp..

[22] Papazoglou A.S., Moysidis D.V., Panagopoulos P., Kaklamanos E.G., Tsagkaris C., Vouloagkas I., Karagiannidis E., Tagarakis G.I., Papamitsou T., Papanikolaou I.G. (2021). Maternal diabetes mellitus and its impact on the risk of delivering a child with congenital heart disease: A systematic review and meta-analysis. J. Matern. Fetal Neonatal Med..

[23] Burlina S., Dalfrà M.G., Lapolla A. (2019). Short-and long-term consequences for offspring exposed to maternal diabetes: A review. J. Matern. Fetal Neonatal Med..

[24] Freud A. (2018). Normality and Pathology in Childhood: Assessments of Development.

